# Quality Initiative for the Administration of Vancomycin Prophylaxis in Penicillin-Allergic Neurosurgery Patients

**DOI:** 10.7759/cureus.18623

**Published:** 2021-10-09

**Authors:** Austin J Borja, Nikhil Sharma, Olivia Amendolia, Jennifer Cimoch, Danielle Callahan, Jennifer Durkan, Nicole Hoke, Eileen Maloney, M. Sean Grady, Neil R Malhotra

**Affiliations:** 1 Neurosurgery, Perelman School of Medicine University of Pennsylvania, Philadelphia, USA; 2 Pharmacy, Hospital of the University of Pennsylvania, Philadelphia, USA; 3 Perioperative Services, Hospital of the University of Pennsylvania, Philadelphia, USA

**Keywords:** vancomycin infusion, surgical site infection, quality improvement, neurosurgery nursing, antibiotic prophylaxis

## Abstract

Introduction

Vancomycin may be used as an alternative perioperative antibiotic for penicillin-allergic patients but follows a different infusion timing. At the institution presented herein, noncompliance with recommended vancomycin infusion timing has been hypothesized to contribute toward increased risk of surgical site infections and avoidable expenditures. The objective of this project was to utilize the Performance Improvement In Action methodology to identify, address, and solve the problem of vancomycin administration timing.

Methodology

This study took place at a multi-hospital, urban academic medical center. The protocol was developed by neurosurgery and anesthesia faculty, advanced practice providers, nursing, and pharmacy. Timing of the following points was recorded: initial order, order release, pharmacy verification, vancomycin infusion, and surgical incision. Fifty consecutive penicillin-allergic patients undergoing neurosurgical intervention were prospectively enrolled. Data comparison was made between the pilot and retrospective review cohorts.

Results

The pilot cohort achieved correct administration of vancomycin in 100% of cases. Average infusion start time prior to incision increased by 257% (p<0.0001).

Conclusions

This study demonstrates a departmental capacity for optimized timing of vancomycin infusions, in a budget- and workflow-neutral process, while reducing inappropriate administration. In the future, this protocol may be scaled to additional departments and institutions to appropriately and efficiently administer perioperative vancomycin and mitigate the risk for surgical site infections.

## Introduction

Surgical site infections (SSIs) have been demonstrated to increase hospital costs and the incidence of patient morbidity and mortality [1]. Perioperative administration of penicillin (PCN) or cephalosporins (cefazolin) is performed as the first-line anti-microbial prophylaxis against SSIs [2]. For patients with a PCN allergy, vancomycin is routinely selected as an alternative perioperative antibiotic [3].

At the university health system studied herein, cefazolin and vancomycin are the most frequently used perioperative antibiotics, accounting for 84% of all procedures performed with antibiotic prophylaxis. National guidelines, such as the Surgical Infection Prevention Project (SIP) and Surgical Care Improvement Project (SCIP), dictate that cefazolin administration should be performed 0-60 minutes prior to surgical incision, while vancomycin administration should occur 60-120 minutes prior to surgical incision [4]. During a retrospective review of 33,457 surgery cases at the studied institution, patients who received cefazolin (n = 26,065) and vancomycin (n = 7,392) were analyzed to determine compliance with the national perioperative antibiotic prophylaxis guidelines [5]. Results of this study concluded that correct administration of cefazolin was 53 times more likely (97% of cases) than correct administration of vancomycin (36% of cases). Further, SSI risk was increased four-fold for vancomycin infusions beginning less than 25 minutes pre-incision. This matched cohort analysis demonstrated a 3.47 million USD increase in hospital costs for patients with SSIs. The authors theorized that the failure to correctly infuse vancomycin was likely a result of impairments in the hospital workflow. Despite numerous evaluations of the impact of vancomycin administration timing, SSIs, and associated costs, there remains a paucity of protocols generated to eliminate inappropriately timed infusions [6-10].

In an effort to enhance the practice of vancomycin administration prior to incision, physician, pharmacy, and nursing leaders initiated a team solution-based approach to improve practice using a Performance Improvement In Action (PIIA) program. The PIIA is a six-phase structured approach to problem-solving, created at the authors’ university health system, to address system issues, discover patient safety opportunities, and improve patient care. The objective of this project was to use the PIIA methodology to develop a protocol to address the problem of vancomycin administration timing and then employ the protocol among a prospective cohort of neurosurgical patients.

## Materials and methods

Context and intervention

The present study reports a multidisciplinary, system-level intervention intended to improve healthcare safety and quality. It adheres to the Standards for Quality Improvement Reporting Excellence (SQUIRE) guidelines [11].

**Figure 1:**
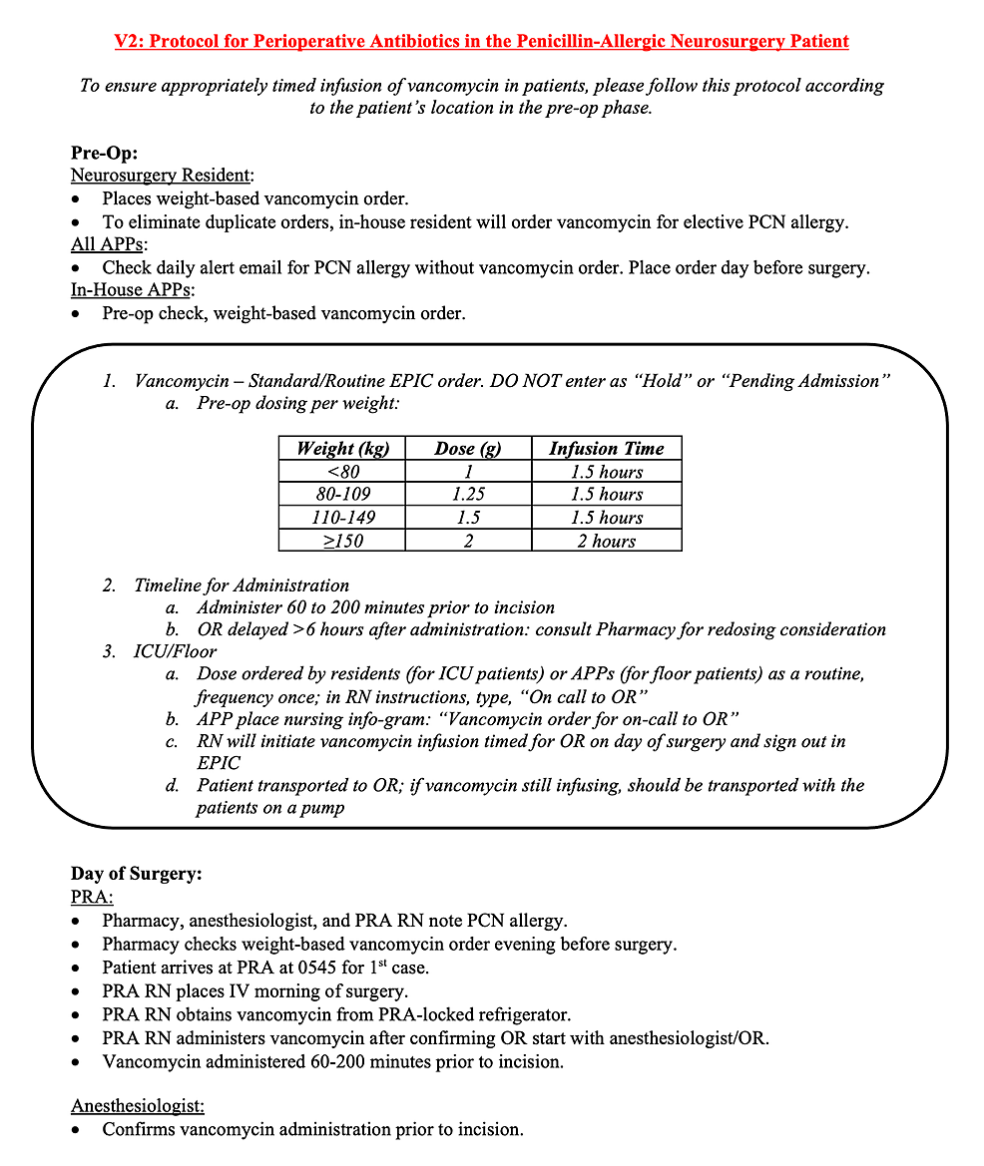
Process Algorithm PCN=Penicillin; APP=Advanced practice provider; OR=Operating room; ICU=Intensive care unit; PRA=Preoperative reporting area; RN=Registered nurse

An SSI elimination protocol was submitted to the university healthcare system institutional review board and accepted as a PIIA project. This protocol was developed by key stakeholders, including neurosurgery faculty, advanced practice providers (APPs), nursing (outpatient, perioperative, and inpatient), anesthesia faculty, and pharmacy. Using this methodology, stakeholders addressed, identified, and worked through the six phases of the PIIA method to identify and propose a solution to the problem of incorrect perioperative vancomycin infusion timing (Figure 1).

During the first phase, Find, an executive sponsor and a facilitator were identified to provide senior-level authority to support, guide, provide resources, and assist teams with removing barriers to protocol success. Next, a team leader and team members were identified to participate in the SSI elimination protocol and attend a two-day classroom experience, weekly meetings, and a two-hour report-out session.

**Figure 2:**
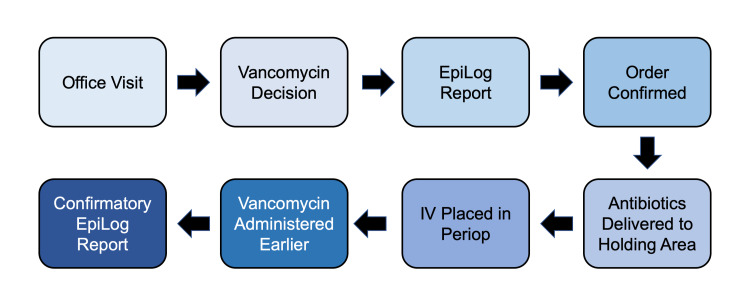
Countermeasures Process Map IV=Intravenous access

During the second phase, Organize and Clarify, the team reviewed and compared the current practice of vancomycin administration to future processes of vancomycin and cefazolin administration. The team identified potential gaps using a process map tool (Figure 2). Process mapping was performed using a smaller cohort of surgical patients, focusing on the neurosurgery patient population.

During the third phase, Understand and Select, the team identified and examined the root cause of the inappropriate timing of vancomycin administration to the perioperative PCN-allergic patient. To do this, inappropriate vancomycin administration data was collected and the related effects of SSIs, length of stay, and hospital costs were reviewed.

During the fourth phase, Plan and Do, the team developed a new workflow to improve intravenous vancomycin administration to PCN-allergic perioperative patients. The team collectively reviewed challenges on the current process and developed two new algorithms to increase vancomycin infusion start time prior to incision. These algorithms are delineated in detail in the next section. Electronic health record (EHR) real-time modification and continual data collection were made possible through the EpiLog tool, a nonproprietary data acquisition software layer into the EHR for quality improvement and cost reduction initiatives with a low cost and minimal impact on employee workflow [12].

Once the algorithms were edited and approved by each of the stakeholders, the team initiated the fifth phase of the PIIA method, Study, and launched the pilot effort to test the new algorithm.

The final step in the PIIA process is Phase six, Act, where measures will be taken to standardize the process and “sustain the gain” of the new protocol. This entails monitoring the metrics of vancomycin administration to ascertain if the process is stabilizing or improving. A Control Plan was established, to demonstrate protocol adherence, or isolate failure, with an action plan for any issue that may arise.

Vancomycin administration algorithms

**Figure 3:**
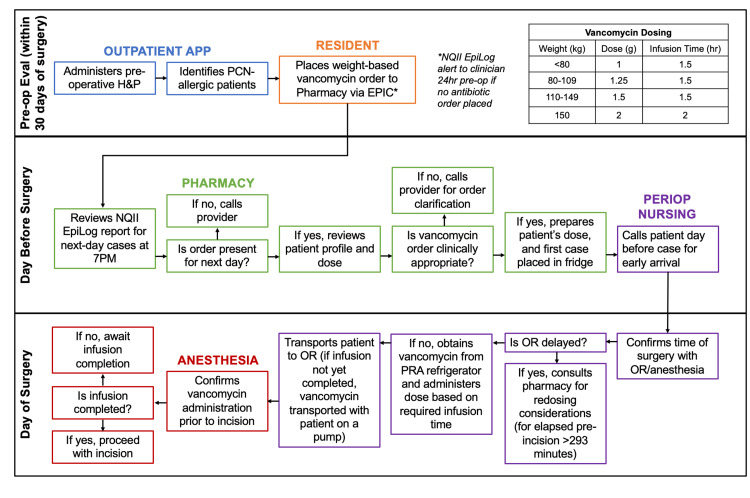
Vancomycin Administration Algorithm for Elective Procedures H&P=History and physical exam; APP=Advanced practice provider; PCN=Penicillin; NQII=Neurosurgery Quality Improvement Initiative; OR=Operating room

The first algorithm was developed for patients who electively scheduled surgery in the outpatient setting (Figure 3). During the preoperative history and physical (H&P) exam, the outpatient APP reviewed the patient’s allergies. If the patient was identified to have a PCN allergy, the outpatient APP communicated this information to the neurosurgery team and ordered an appointment for the patient to be seen by Allergy medicine. Following positive sensitivity testing by Allergy specialists, the patient was then confirmed as PCN-allergic. To optimize this process and ensure this step was not missed, there was an alert built into the documentation of the H&P, notifying the outpatient APPs of potential PCN-allergic patients. Outpatient APPs also worked with the pharmacist to confirm the accuracy and timeliness of vancomycin orders. The Neurosurgery resident physician was responsible for placing the weight-based vancomycin order for the outpatient and intensive care unit (ICU) PCN-allergic patients.

**Figure 4:**
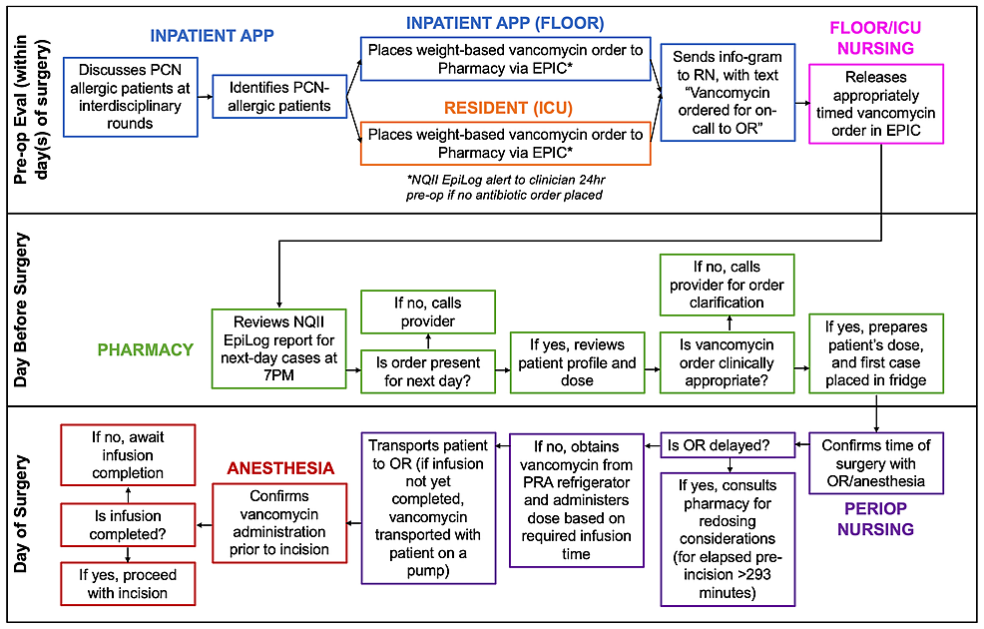
Vancomycin Administration Algorithm for Inpatient Procedures APP=Advanced practice provider; PCN=Penicillin; ICU=Intensive care unit; NQII=Neurosurgery Quality Improvement Initiative; OR=Operating room

The second algorithm was developed for patients who were scheduled for surgery in the inpatient setting (Figure 4). In the day(s) prior to surgery, inpatient APPs identified PCN-allergic patients and placed a weight-based vancomycin order for the neurosurgical in-patient unit. To ensure this step was not missed, a “pre-op vancomycin order for PCN-allergic patients” check box was added to the previously established and operationalized pre-op checklist. The inpatient APP was responsible for communicating to the bedside nurse through sending a nursing info-gram reminder, in an effort to ensure the order was seen. Finally, during morning interdisciplinary care rounds, the inpatient APP and nursing leadership reviewed all PCN-allergic patients on the operating room schedule to ensure both that all PCN-allergic patients were identified and had received preoperative vancomycin infusions. For both algorithms, the Pharmacist reviewed an automated daily vancomycin report. This was performed by accessing the “Surgical Orders Requiring Advanced Preparation” report to find neurosurgical PCN-allergic patients who had vancomycin orders entered at the pre-operative visit. To ensure the vancomycin order was present for next-day surgeries, the pharmacist verified the correct dose, prepared, and delivered vancomycin to the refrigerator in the Preoperative Reporting Area (PRA) (for outpatient procedures) or floor/ICU (inpatient procedures) the night before or day of surgery. If the order was not in the advanced preparation report or the dose was incorrect, the pharmacist would email the vancomycin timing group for a provider to place/modify the order. The perioperative nurse would call first-case patients the night before their surgery to ensure their timely arrival the next morning. On the day of surgery, the perioperative nurse confirmed the time of surgery, placed an intravenous catheter, and administered the vancomycin dose (60 to 120 minutes prior to incision). Patients needed to be brought in at least 90 minutes prior to the scheduled procedure time so the infusion could begin within the required timeframe. In addition to the time adjustment, the pre-op unit needed to coordinate with the pharmacy and neurosurgery service to have the vancomycin doses available at the appropriate time. Further, it was imperative that the perioperative nurses confirmed the surgery time with the operating room staff or anesthesiologist before starting the infusion, in case of a delayed start time. Prior to incision, the anesthesiologist confirmed and documented that vancomycin had been fully administered.

Study of the intervention

The index cohort retrospectively, previously described, enrolled all patients undergoing surgical intervention across the present multi-hospital, university health system, from November 1, 2014, to October 31, 2015, who received perioperative vancomycin administration for SSI prophylaxis (n = 7,392) [5]. Data on preoperative antibiotic administration, surgery scheduling, and postoperative infection were acquired from the EHR.

For the pilot cohort (PIIA cohort), all consecutive elective surgical patients from March-June 2017 were screened (n = 508). Of these, all PCN-allergic patients undergoing neurosurgical intervention and determined to require perioperative vancomycin infusion (n = 50) were prospectively enrolled. During the pilot study, the date and time were recorded for the following points: initial order, order release, pharmacy verification, vancomycin infusion (start and finish), and surgical incision.

The primary outcome measure was the proportion of patients with correct vancomycin infusion timing. Secondary measures included average infusion start time prior to incision and failure rate of administration during the critical infection window (characterized by an infusion start time of 30 minutes or less prior to incision). The efficacy of the revised vancomycin infusion protocol was compared between the PIIA cohort and the index cohort. Chi-squared test was employed to compare categorical variables, while Wilcoxon rank-sum test was performed to compare continuous measures.

Ethical considerations

All subjects consented to involvement in the prospective study portion, which was approved by the institutional review board (IRB) at the present university. This study was considered exempt from approval by the IRB as this is considered a quality improvement study. All ethical guidelines and rules were followed to protect patient privacy. The Bernadette and Kevin McKenna Neurological Surgery Research Fund provided critical salary support for research assistants and statistical analysis. The authors declare no conflicts of interest.

## Results

After the algorithms were implemented into the neurosurgical workflow, the PIIA cohort (n = 50) achieved correct administration of vancomycin in 100% of cases, a 178% increase (p < 0.0001) from the index cohort.

**Figure 5:**
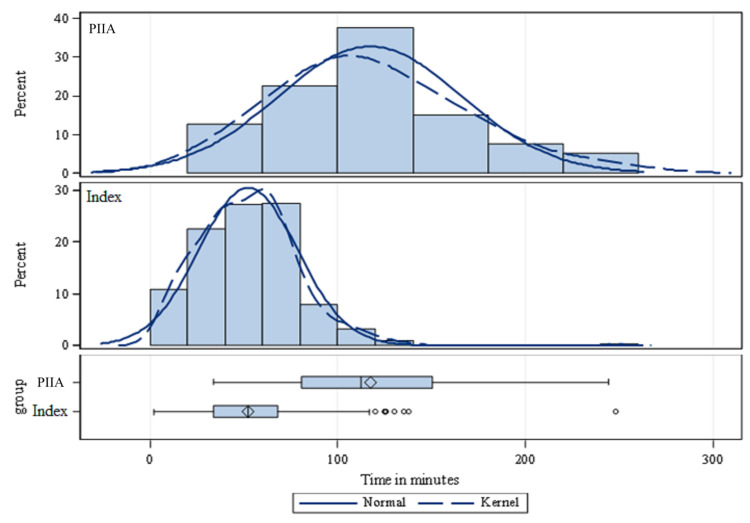
Normalized Graphs Comparing Pre-Incision Infusion Start Times for the PIIA and Prior Cohorts (Index) PIIA=Performance Improvement In Action

The average infusion start time prior to incision increased by 257% (p < 0.0001), from 45.6 minutes (index cohort) to 117.1 minutes (PIIA cohort) prior to incision. Normalizing data from both the index cohort and the PIIA cohort depicts this increase in infusion start time (Figure 5).

**Figure 6:**
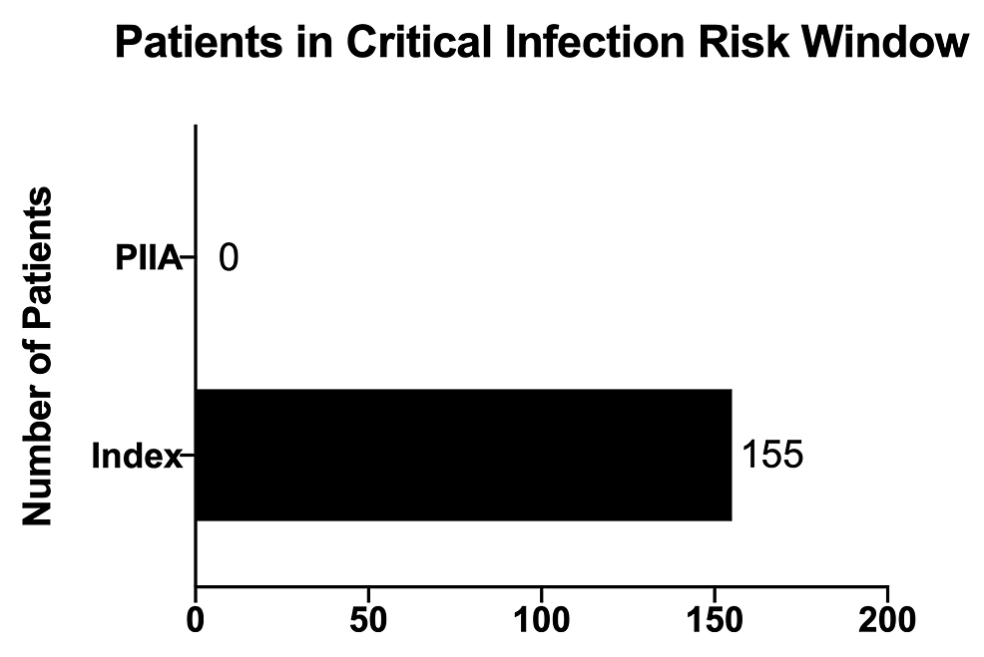
Number of Patients in the Critical Infection Window PIIA=Performance Improvement In Action

Additionally, the failure rate of administration during the critical infection window was eliminated (Figure 6).

## Discussion

This study demonstrates how quality improvement frameworks can be utilized to actively enhance care delivery. The implication at the studied institution has been a continuation of the established protocol of vancomycin delivery, which has sustained infection rates at a near-0% rate in the time since pilot study completion. Our multi-disciplinary approach to using quality improvement frameworks to solve patient care delivery issues can be translated across medical and surgical specialties.

Workflow optimization in healthcare has become increasingly possible due to new technology, an expanding array of health professionals and care teams, and initiatives to improve patient safety and make care more patient-centered [13]. The PIIA model used in this study is a six-phase systematic approach to efficiently and effectively improve existing processes at the authors’ institution. By utilizing the PIIA method, multi-disciplinary leaders were able to identify systemic problems, establish a team of stakeholders to address these issues, and construct a process to successfully implement a solution, thus resolving the matter.

Although there was a 100% success rate of correct administration using this new algorithm, the change in workflow presented numerous challenges to the parties involved. The new vancomycin ordering process within the EHR presented as one of the primary obstacles. This was due, in large part, to the learning curve with the hospital’s transition to Epic (Epic Systems Corporation, Madison, WI, USA), as this system has different functionality than the previous electronic ordering system. Issues arose with respect to determining the proper weight-based antibiotic dose and with the designated person placing the order in a timely fashion. Lag time is to be expected when implementing new protocols; however, this issue was compounded by the fact that Epic did not prevent duplicate orders of vancomycin from being sent to the pharmacy. This presented challenges for both those placing and receiving the vancomycin order.

Orders for outpatients required providers to enter vancomycin orders through a virtual portal, which then appeared on a “Surgical Orders Requiring Advanced Preparation” report for the pharmacy. From a pharmacy standpoint, the biggest issue with the virtual portal was the limitation in functions that the pharmacist was able to perform (e.g., dose changes and rejecting incorrect or duplicate orders). Of these limitations, the ability to reject duplicate orders was most concerning, as this created the potential for the patient to be over-dosed if the error went unnoticed. In addition, pharmacist adjustment requests to the outpatient provider did not always result in timely intervention prior to the patient’s procedure. In cases where the dose needing adjustment was not corrected the night prior to the procedure, administrative and operational delays increased.

Another issue was the potential for multiple orders being prepared and administered to the patient, in cases where multiple orders were entered. Inpatients were at similar risk as outpatients, minus the virtual portal, but with the additional challenge of order administration not being released until the appropriate time. For inpatients, floor or ICU nurses were tasked with releasing orders within a timeframe that allowed for proper vancomycin administration. Unfortunately, there were a number of instances where the order was placed, but the nursing staff failed to release the order to the pharmacy. On a few occasions, when an order was released in Epic for a first start case, the time erroneously defaulted to midnight in Epic. If the mistake was not detected by pharmacy, this could have led to premature delivery and antibiotic administration.

For NICU nurses, there were certain barriers that made adhering to the new protocol challenging. Given that patients in the NICU are critically ill, emergency surgeries are more common for NICU patients than patients on the floor. On the occasion that emergency surgery is necessary, it is often scheduled only hours or minutes prior to incision time. This short time period is often not conducive for allowing adequate time for vancomycin prior to incision.

Herein, our objective was to implement a novel protocol to address the problem of inappropriately timed vancomycin infusion prior to neurosurgery, and we achieved 100% correctly timed administration in our pilot cohort. For this study, we did not intend to assess adverse effects associated with vancomycin. As such, for the present work, we did not have the full and complete data to report adverse pharmacological effects, such as infusion reaction, anaphylaxis, acute kidney injury, ototoxicity, or Stevens-Johnson syndrome/toxic epidermal necrolysis, subsequent to infusion [14-17]. While we do not anticipate the incidence of these side effects to differ substantially between the PIIA and index cohorts, physicians administering vancomycin should anticipate and promptly respond to these debilitating and potentially life-threatening complications. Moreover, at the present institution, a separate, dedicated study is being pursued to characterize vancomycin-related side effects within our population.

Currently, this process is in the “Study and Act” steps of the PIIA, where the team is investigating how the process has impacted patients and workflow. A potential next step for this work is creating a replication plan, which would allow other departments or hospitals to adopt this protocol into their practice. In its simplest form, this protocol helps to reduce the inappropriate administration of vancomycin to patients who are PCN-allergic, thus aiding in reducing the risk for SSIs. The department herein was able to achieve this in a budget- and workflow-neutral fashion. The authors strongly believe that this protocol can be generalized to offer significant benefits to other departments and institutions to curb the risk for SSIs, and more importantly, plan and administer perioperative vancomycin appropriately and efficiently.

Limitations

There were several limitations associated with this study. First, the PIIA cohort consisted of a relatively small sample size (n = 50). Next, there were occasional errors in the EHR alert system, which caused the administration of multiple doses of vancomycin. Although this problem was fixed, the process is undergoing continual efforts to make the alert system error-free. Further, implementing the new protocol required a workflow change in the clinic setting, where preoperative order planning is critical, as well as in the PreOp unit, where nursing took on the additional responsibility of IV placement and initiating vancomycin infusions. Additional impacts of implementing the new algorithm included the potential need for the patient to arrive earlier to begin infusion and a prolonged PreOp bed utilization for PCN-allergic patients. Finally, it remains possible that the implementation of this new protocol may bias providers because of increased attention on the problem identified herein. Nevertheless, the achievement of correct vancomycin administration timing across the PIIA cohort remains compelling.

## Conclusions

Appropriately timed antibiotic infusions are critical to reducing the risk of SSIs. At the present institution, noncompliance with recommended vancomycin infusion timing has previously been associated with an increased risk of SSI and increased hospital expenditures. However, there is a paucity of protocols that have been developed and published for the elimination of inappropriately timed infusions. This study demonstrates a departmental capacity for optimized timing of vancomycin infusions, in a budget- and workflow-neutral process. A multidisciplinary problem-solving approach enabled the creation of an algorithm that incorporated each stakeholder’s point of view and responsibility in the process. Under this protocol, inappropriate administration of vancomycin was eliminated in the pilot cohort. The present institution has subsequently continued this established protocol and maintained near-zero infection rates since the completion of this study. Moving forward, this pilot should be brought to scale in the studied center, and other institutions, to continue efforts to reduce SSIs in surgical patients.
